# Comprehensive transcriptome analysis of *Sarcophaga peregrina*, a forensically important fly species

**DOI:** 10.1038/sdata.2018.220

**Published:** 2018-11-06

**Authors:** Ji Yeon Kim, Hye Young Lim, Sang Eon Shin, Hyo Kyeong Cha, Jeong-Han Seo, Suel-Kee Kim, Seong Hwan Park, Gi Hoon Son

**Affiliations:** 1 Department of Biomedical Sciences, College of Medicine, Korea University, Seoul 02841, Korea; 2 Department of Legal Medicine, College of Medicine, Korea University, Seoul 02841, Korea; 3 Research and Development Center, Insilicogen Inc., Yongin 16954, Gyeonggi-do, Republic of Korea

**Keywords:** Transcriptomics, Entomology

## Abstract

*Sarcophaga peregrina* (flesh fly) is a frequently found fly species in Palaearctic, Oriental, and Australasian regions that can be used to estimate minimal postmortem intervals important for forensic investigations. Despite its forensic importance, the genome information of *S. peregrina* has not been fully described. Therefore, we generated a comprehensive gene expression dataset using RNA sequencing and carried out *de novo* assembly to characterize the *S. peregrina* transcriptome. We obtained precise sequence information for RNA transcripts using two different methods. Based on primary sequence information, we identified sets of assembled unigenes and predicted coding sequences. Functional annotation of the aligned unigenes was performed using the UniProt, Gene Ontology, and Kyoto Encyclopedia of Genes and Genomes databases. As a result, 26,580,352 and 83,221 raw reads were obtained using the Illumina MiSeq and Pacbio RS II Iso-Seq sequencing applications, respectively. From these reads, 55,730 contigs were successfully annotated. The present study provides the resulting genome information of *S. peregrina*, which is valuable for forensic applications.

## Background & Summary

Forensic entomology is an invaluable tool for estimating minimal postmortem interval (PMI) in criminal investigations. For the precise estimation of PMI, it is necessary to identify species and developmental stages of insects in corpses^1–3^. Some arthropods, particularly those belonging to the order Diptera (flies), are attracted to the bodies of dead animals. Flesh flies from the Sarcophagidae family usually appear on corpses slightly later than blow flies from the Calliphoridae family and are considered the second-most important species for forensic applications^4–7^. Flesh fly larvae are usually larger than Calliphoridae larvae at the same developmental stage, making them more conspicuous at the death scene and easier to sample by investigators^8^. The minimal PMI is usually estimated based on the developmental stage of the oldest larvae by calculating accumulated degree hours (ADH)^8^.

*Sarcophaga peregrina* (*S. peregrina*) is widely distributed in Palaearctic, Oriental, and Australasian regions^9^. It is found in many insect succession studies and at many death scenes^10–14^. In a previous study comparing insect succession patterns in human cadavers and animal carcasses, *S. peregrina* was found on human, pig, and rabbit corpses^13^. Importantly, *S. peregrina* was found in 7 out of 35 (20%) medicolegal autopsy cases in the northeastern area of Seoul, Korea^15^ and in 16 out of 42 (38%) autopsy cases in Saitama Prefecture, Japan^16^. *S. peregrina* is also related to parasitic diseases such as myiasis in both human and livestock, which is often forensically important as it can imply abuse^17,18^. Despite its forensic and medical significance, genomic information for *S. peregrina* has not yet been described.

**Figure 1:**
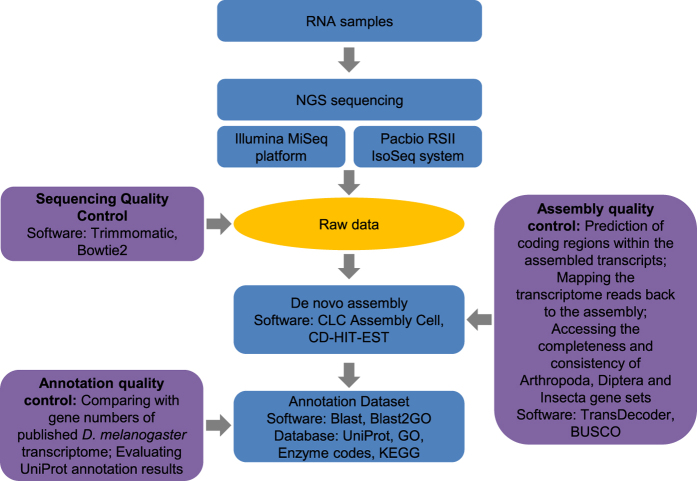
Schematic overview of the study. Samples were prepared by pooling equal amounts of RNA from each developmental stage of *S. peregrina*, including early-, middle-, and late-instar larvae; middle-stage pupae; and adults. cDNA was synthesized and sequenced using the Illumina MiSeq platform with paired-end reads. For more accurate gene prediction of *S. peregrina*, we also employed the Pacbio RS II Iso-Seq system for full-length transcript sequencing. Analysis started with the assembly of full-length transcripts and corresponding isoforms using the *de novo* assembly program CLC Assembly Cell without a reference genome. The sequenced transcripts derived from Iso-Seq and MiSeq were combined, and the CD-HIT-EST program was used to construct the final standard transcript to eliminate redundancy. To examine the completeness of *S. peregrina* unigenes, we used the TransDecoder program BUSCO analysis and continued with functional analysis using the BLAST program. Quality control assessments were performed at each step.

In the present study, we carried out *de novo* transcriptome assembly in *S. peregrina* using high-throughput RNA sequencing (RNA-seq) followed by bioinformatic gene modeling. The assembled contigs were annotated, thereby enabling generation of the first gene catalogs for *S. peregrina*. The catalog of annotated *S. peregrina* genes in this study improves upon currently available dipteran transcriptome datasets and is the first comprehensive analysis of gene expression profiles for *S. peregrina*. Our data could be applied to RNA-based studies on this forensically important fly species including molecular approaches to the growth and development of *S. peregrina* for forensic investigations. Overall experimental workflow is summarized in Fig. 1.

## Methods

### Sample collection

We established a breeding colony from wild-type *S. peregrina* flies collected in the Northeastern region of Seoul, Korea. Flies were kept in a temperature-controlled chamber [24±0.8°C, 70±5% relative humidity, and 16:8 h (light:dark) photoperiod]. Specimens were prepared from F3 progeny at the following five developmental stages: early- (n = 30), middle- (n = 5), and late-instar larvae (n = 3); middle-stage pupae (n = 3); and newly-emerged adults (2 males and 2 females). We confirmed species identities of our specimens using nucleotide sequences of mitochondrial cytochrome c oxidase subunit I (COI) as described in our previous study^15^. Developmental times were quantified as a sequence of days and ADH using a developmental threshold temperature of 10.9°C^19,20^. A total ADH value spanning all five stages from egg to adult was calculated as 5,895 h. The collected larvae were observed under an Olympus SZX10 stereomicroscope (Olympus, Japan) to determine the larval instar based on the number of clefts in the posterior spiracle. The pupal stage was observed at a 4- or 8-h interval until adult eclosion. The wandering duration, pupation, and eclosion time points were recorded during the experiment. Whole body samples were quickly frozen in liquid nitrogen and stored at −70 °C for subsequent RNA extraction.

### RNA preparation

Each sample was homogenized with liquid nitrogen in a mortar. Total RNA was extracted using a RNeasy mini kit (Qiagen GmbH, Hilden, Germany) according to the manufacturer’s instructions. Equal amounts of RNA samples from different developmental stages (10 μg per sample) were pooled for RNA sequencing. RNA concentration was assessed using a NanoDrop™ spectrophotometer (Thermo Fisher Scientific, Waltham, USA), and RNA integrity number values were calculated by an Agilent 2100 Bioanalyzer (Agilent Technologies, Santa Clara, USA).

### Library construction and sequencing

We purified poly(A) mRNA using Oligo (dT) magnetic beads (Qiagen), and the purified mRNA was broken into short fragments. Double-stranded cDNA was synthesized with sequencing adapters using the TruSeq^TM^ Stranded mRNA prep Kit (Illumina). Finally, library sequence data were acquired using paired-end sequencing via the Illumina MiSeq platform. For more accurate gene prediction of *S. peregrina*, Iso-Seq using the Pacbio RS II system was employed for full-length transcript sequencing. Library construction and sequencing processing were conducted following the manufacturer’s instructions. Raw reads from MiSeq paired-end sequencing underwent pre-processing by removing Illumina TruSeq^TM^ adapter sequences and low-quality sequences (<Q20) using trimmomatic^21^ with default parameters. To identify contaminant sequences for removal, clean reads without adapter and low-quality bases were mapped to bacterial and ocean metagenome databases downloaded from NCBI (ftp://ftp.ncbi.nlm.nih.gov/genomes/Bacteria, ftp://ftp.ncbi.nlm.nih.gov/genomes/Bacteria_DRAFT, ftp://ftp.ncbi.nlm.nih.gov/genomes/all/GCA/000/204/965/GCA_000204965.1_ASM20496v1) using the default setting of bowtie2. Those regions that did not map to the databases were subsequently removed from further analysis.

### *De novo* assembly and dataset annotation

High-quality sequences were used in the subsequent assembly. Transcripts were assembled using CLC Assembly Cell (version 5.0; CLC bio, Waltham, MA, USA), which is optimized to present the best *de novo* results compared with a variety of assemblers that utilize MiSeq paired-end reads. Transcripts derived from Iso-Seq and Miseq were combined, and the CD-HIT-EST program (version 4.6.5)^22,23^ was used to construct the final standard transcript with default parameters (similarity 95%) to eliminate transcript redundancy. The coding region prediction of assembled transcripts was performed using TransDecoder (version 3.0.0; implemented in Trinity software) (http://transdecoder.github.io). We then assessed its overall inclusiveness/completeness by performing a benchmarking universal single-copy orthologs (BUSCO) analysis (https://busco.ezlab.org/)^24^. We used OrthoDB database of orthologs (www.orthodb.org) to define BUSCO sets for three major phylogenetic clades, which use 1,066 for Arthropoda, 2,799 for Diptera and 1,658 for Insecta near-universal single-copy orthologs. The Blast2GO program (E-value < 1e^-^3) was used to annotate the unigenes based on UniProt (http://www.uniprot.org/help/uniref) and Kyoto Encyclopedia of Genes and Genomes (KEGG) databases (http://www.genome.jp/kegg/). Gene Ontology (GO) terms were assigned to each unigene based on the GO terms annotated to its corresponding homologs in the UniProt database. The obtained contigs of *S. peregrina* were then analyzed by gene family identification for annotation quality control. The following pipeline was used to cluster individual genes into gene families: (i) *S. peregrina*-to-*D. melanogaster* blastp was used to align all protein sequences with an E-value of 1e^-^3, and (ii) the gene families were clustered using OrthoMCL software^25^. Protein sequences of *D. melanogaster* were downloaded from Ensembl (release 85).

## Data Records

Three types of datasets were generated in this study. The first dataset consists of RNA-seq raw reads of *S. peregrina,* which were submitted to the NCBI database (Data Citation 1 and Table 1). The second dataset contains the unigenes of *S. peregrina* (Data Citation 2 and Table 2). The third dataset comprises the annotation results for all databases and the predicted coding regions (CDSs) and protein information (Data Citation 3 and Table 3). This dataset also contains OrthoMCL results from the complete proteome of *S. peregrina* and *D. melanogaster* for examining transcript annotation completeness (Data Citation 3 and Table 3).

## Technical Validation

### Sequencing quality control

We evaluated sequencing quality to determine whether our results sufficiently cover the transcriptome of *S. peregrina*. A total of 26,580,352 raw reads were obtained by Illumina MiSeq platform paired-end sequencing. However, this conventionally applied short-read sequencing platform does not reliably distinguish many transcript isoforms. For more accurate gene prediction, we additionally employed Pacbio RS II Iso-Seq for full-length transcript sequencing. A total of 83,221 raw reads were obtained by full-length transcript sequencing (Table 4). We also tested samples using FastQC^26^ for Q20 and GC content (Table 4).

### Assembly quality control

Trimmomatic^21^ was used to improve the overall quality of the assembly by removing adaptor contamination and serving as a quality control assessment tool for raw reads. Clean reads derived from bacterial and viral genomes were mapped to the bacterial and the ocean metagenome databases downloaded from NCBI by applying bowtie2. *De novo* assembly of the clean reads was performed using CLC Assembly Cell, which reconstructs full-length transcripts and corresponding isoforms without a reference genome. Transcripts derived from Iso-Seq and MiSeq were combined, and the CD-HIT-EST program was used to construct the final standard transcript with default parameters (similarity 95%) to eliminate transcript redundancy. After assembly and combination, 77,089 contigs were obtained. These contigs contain many isoforms. Coding-region prediction in the assembled transcripts was performed using the TransDecoder program implemented in Trinity software to examine the completeness of *S. peregrina* unigenes. As a result, 55,730 unigenes were identified by open reading frame prediction in the entire transcriptome of *S. peregrina*. The contig lengths of *S. peregrina* ranged from 237 to 13,704 bp with an average length of 623.43 bp (Table 5). The *S. peregrina* assemblies were also evaluated using the BUSCO arthropod, Diptera and Insecta gene sets, which use 1,066, 2,799 and 1,658 near-universal single-copy orthologs to assess the relative completeness of genome and transcriptome assemblies. The percentage of conserved genes identified in the *S. peregrina* assembly compares favorably with metrics reported for a number of insect transcriptomes and model insect genome assemblies (Table 6).

### Annotation quality control

**Figure 2:**
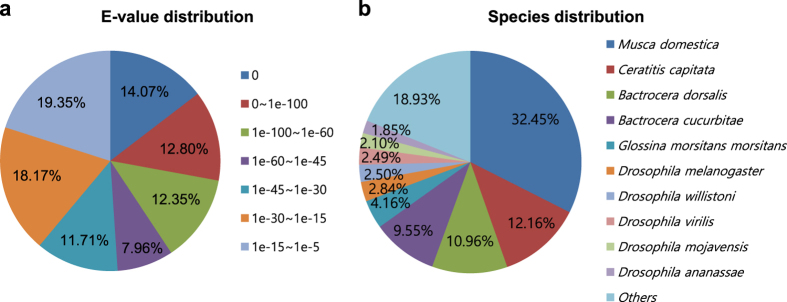
Characteristics of homology search of contigs against the UniProt protein database. (**a**) E-value distribution of the top BLAST hits for each contig (E-value<1.0 e^-^3). (**b**) Hit species distribution.

**Figure 3:**
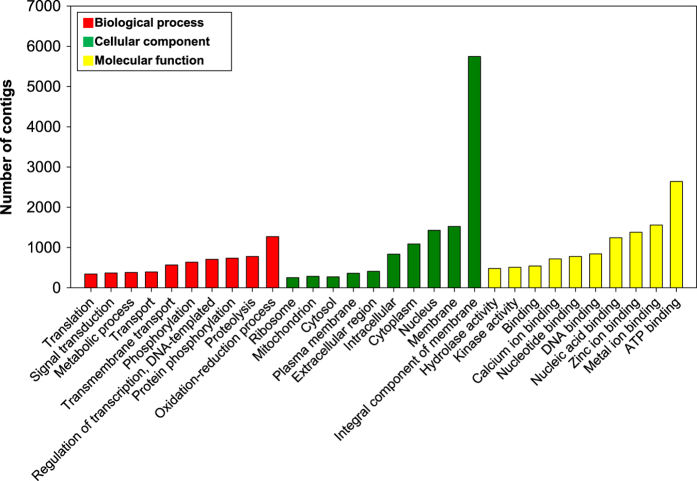
GO classification. Results are summarized in three main categories: biological process, cellular component, and molecular function. The y-axis indicates the number of contigs.

We estimated functional annotation results based on the aforementioned database and detailed information from the UniProt database (Table 7), which revealed that 33,991 unigenes (60.99%) were aligned to the UniProt database. The E-value distribution of the top hits showed that 39.22% of the sequences have strong homology (smaller than 1e^-^60) (Fig. 2a). Also, the top hit species distribution of matches with known sequences indicates that the majority of *S. peregrina* sequences show the highest homology with *Musca domestica* sequences (32.44%). The other most-represented species include insects like flies (Fig. 2b). All alignments were carried out using E-value thresholds of <1e^-^3. Next, Blast2GO was used to assign GO terms and functionally categorize the assembled *S. peregrina* contigs. Many of the assembled contigs correspond to at least one GO term (23,269 contigs, 41.75% of all contigs; Fig. 3). The annotated transcript sequences represent a significant contribution to the genomic information available for this species.

To identify the biological pathways active in *S. peregrina*, we mapped the 55,730 annotated sequences to reference canonical pathways in KEGG. A total of 6,335 unigenes (11.36%) were assigned to 132 known metabolic or signaling KEGG pathways (Table 7). Next, we applied OrthoMCL to the complete proteome of *S. peregrina* and *D. melanogaster*. From the dataset of 86,092 proteins (30,362 from *D. melanogaster* and 55,730 from *S. peregrina*), OrthoMCL categorized 37,365 proteins (14,584 for *S. peregrina* and 22,670 for *D. melanogaster*) into 8,378 groups (E-value < 1e^-^3). These results show that orthologs between two species can be identified using our data. There were 1.74 and 2.70 orthologs in the average ortholog group of *S. peregrina* and *D. melanogaster* (Table 8).

## Usage Notes

The data provided in these experimental datasets can be used for two purposes. First, the raw reads can be used to conduct new analyses using different methods. Second, each analysis step can be repeated with specific technical changes, as all technical and experimental information is publicly available.

### *De novo* assembly

The *de novo* assembly of RNA sequencing reads without a reference genome remains a challenge despite the development of many bioinformatic tools for data assembly and analysis^27–30^. In this study, a reference transcriptome for *S. peregrina* was sequenced and annotated using Illumina MiSeq and PacBio Iso-Seq sequencing technologies obtaining 7872.2 Mbp and 191.3 Mbp of transcriptome data, respectively, which further assembled into 55,730 unigenes. To the best of our knowledge, this is the first study to obtain whole transcriptome information using RNA sequencing in *S. peregrina*. In the past few years, interest in the forensic investigation of *S. peregrina* has increased in many countries. The limited genetic information and lack of understanding of the molecular mechanisms pertaining to *S. peregrina* development and reproduction have been the main obstacles preventing further studies. Our study provides the most extensive sequencing resource and genetic information of *S. peregrina* available to date, which provides a foundation for further molecular studies. Whole transcriptome data of *S. peregrina* were obtained using high-throughput sequencing, and unigenes were annotated using three main databases. Taken together, these results provide a solid foundation for further research on *S. peregrina* at the molecular level and can serve as an important genomic tool for forensic entomology communities.

### Downstream analysis

The present study can be utilized as a reference for a variety of forensic entomological studies requiring RNA sequence information of *S. peregrina* and related species. For example, considering that *S. peregrina* belongs to necrophagous flies developing on corpses, identification of developmental stage-specific gene transcripts may greatly contribute to entomological approaches to PMI estimation. Determining the age of juvenile necrophagous flies is one of the key tasks in forensic entomology to provide evidence for the minimal PMI. As the age determination of the necrophagous fly larvae has largely relied on using morphological parameters, it is quite difficult to estimate the progressing pupal stage, which lasts about half of the total juvenile development without apparent morphological changes. In this regard, several previous studies have tried to identify sets of genes exhibiting a developmental stage-specific expression profiles in flesh flies by differential display^31^ or subtractive hybridization methods^32^. However, more systemic and genome-wide analyses on development-related gene expression in the flesh flies have not been yet carried out primarily due to lack of sufficient genomic information. The genome-wide transcriptome information and subsequent examination of development-associated expression profiles will provide novel molecular biomarkers for forensic applications of the *S. peregrina*. Identification of RNA species with highly developmental stage-specific expression profiles, in particular for pupal stages will greatly contribute to rapid and precise determination of the developmental stages, thereby estimation of the minimal PMI. To break new ground in the field of age determination of forensically relevant flies, additional transcriptome analysis would be required for each developmental stage. Data obtained in this study will serve as a basis to establish molecular age determination techniques based on *S. peregrina* and other necrophagous flies.

## Additional information

**How to cite this article:** Kim. J. Y. *et al*. Comprehensive transcriptome analysis of Sarcophaga peregrina, a forensically important fly species. *Sci. Data*. 5:180220 doi: 10.1038/sdata.2018.220 (2018).

**Publisher’s note:** Springer Nature remains neutral with regard to jurisdictional claims in published maps and institutional affiliations.

## Supplementary Material



## Figures and Tables

**Table 1:** Raw data deposit.

Sample no.	SRA Runs	BioSample	Title
1	SRR6265701	SAMN07981104	Peregrina-Pooled-RNA_1.fastq and Peregrina-Pooled-RNA_2.fastq
2	SRR6265702	SAMN07981103	NIR_all_quivered_hq.100_30_0.99.fastq
The dataset consists of two samples. Sample 1 is from the paired-end sequencing dataset obtained using the Illumina MiSeq platform. Sample 2 is from the full-length sequencing dataset obtained using the Pacbio RS II system. Sequence data were deposited in the Sequence Read Archive (SRA, accession numbers SRR6265701 and SRR6265702) (Data Citation 1).			

**Table 2:** Unigene deposit.

File name	File type	Data
SPER_Unigenes	fasta	unigenes
The dataset contains unigenes from the longest contigs per transcript generated using the CLC Assembly Cell, CD-HIT-EST program. The SPER_Unigenes file contains total unigenes from *S. peregrina*. The unigene file was deposited into the Transcriptome Shotgun Assembly Sequence database (accession number GGEP00000000) (Data Citation 2).		

**Table 3:** Annotation deposit.

File name	File type	Data description
SPER_blast2go_GO	Xls	GO database annotation
SPER_blast2go_kegg	Xls	KEGG database annotation
SPER_blast2go_uniprot	Xls	UniProt database annotation
SPER_denovo_Transcriptome_CDS	fasta	Predicted coding sequence
SPER_Transcriptome_protein	fasta	Predicted protein sequence
DMEL_SPER_ortholog_genes	Xls	Orthlog gene annotation
The dataset contains functional annotations and gene coding sequence annotations for *S. peregrina*. There are five annotation files, three of which are functional annotation files and two of which are structural annotation files. The three functional annotation files are the GO, KEGG, and UniProt database annotation files. The sequence annotation files are in fasta format; the titles in the files contain the unigene name predicted coding sequence, locus, and coding direction. This dataset also contains OrthoMCL results from the complete proteome of *S. peregrina* and *D. melanogaster* for examining transcript annotation completeness. The annotation file is available in the Figshare database (Data Citation 3).		

**Table 4:** Quality control and data statistics of the raw reads.

Type	MiSeq	Iso-Seq
Read number	26,580,352	83,221
Read length (Mb)	7,872.2	191.3
Q20 (%)	90.42	NA
GC (%)	38.14	36.10

**Table 5:** Assembly statistics.

Type	*S. peregrina*
Total numbers of unigenes	55,730
Total numbers of transcripts	77,089
Total length (bp)	34,742,946
N50 (bp)	1,245
Average length (bp)	623.43
Max length (bp)	13,704
Min length (bp)	237
GC (%)	39.87

**Table 6:** BUSCO analysis of assembly completeness.

BUSCO results	Arthropoda		Diptera		Insecta	
Complete BUSCOs	970	90.99%	2,112	75.46%	1466	88.42%
Complete single-copy BUSCOs	718	67.35%	1,446	51.66%	1062	64.05%
Complete Duplicated BUSCOs	252	23.64%	666	23.79%	404	24.37%
Fragmented BUSCOs	58	5.44%	424	15.15%	112	6.76%
Missing BUSCOs	38	3.56%	263	9.40%	80	4.83%
Total BUSCO groups searched	1,066	100%	2,799	100%	1,658	100%

**Table 7:** Annotation statistics.

Type	*S. peregrina*
Unigene number	55,730
UniProt	33,991
GO	23,269
KEGG	6,335

**Table 8:** Ortholog groups of *S. peregrina* and *D. melanogaster* identified by OrthoMCL.

Organism	Total proteins	Orthologs	Ortholog groups	Specific genes (no blast+specific paralog)	Extra (with blast, no grouping)	Orthologs/ortholog groups
*S. peregrina*	55,730 (100%)	14,584 (26.17%)	8,378	7,463 groups (1,685 + 12,435)	27,026 (48.49%)	1.74
*D. melanogaster*	30,362 (100%)	22,670 (74.67%)	8,378	2,250 groups (132 + 5,350)	2,210 (7.28%)	2.70
